# Supplementary material for: Modeling Patient-Specific CAR-T Cell Dynamics: Multiphasic Kinetics via Phenotypic Differentiation
Source: Cancers (Basel). 2022 Nov 14;14(22):5576. doi: 10.3390/cancers14225576 (PMC9688514; doi:10.3390/cancers14225576)
Supplement: Supplementary file 1 [file cancers-14-05576-s001.zip › cancers-1958701-sm.pdf]

cancers

MDPI

Article

Modeling Patient-Specific CAR-T Cell Dynamics: Multiphasic Kinetics via Phenotypic Differentiation

Emanuelle A. Paixão [1,*], Luciana R. C. Barros [2], Artur C. Fassoni [3] and Regina C. Almeida [4]

1 Graduate Program, Laboratório Nacional de Computação Científica, Petrópolis 25651-075, Brazil
2 Center for Translational Research in Oncology, Instituto do Câncer do Estado de São Paulo, Hospital das Clínicas da Faculdade de Medicina da Universidade de São Paulo, São Paulo 01246-000, Brazil
3 Institute for Mathematics and Computer Science, Universidade Federal de Itajubá, Itajubá 37500-903, Brazil
4 Computational Modeling Department, Laboratório Nacional de Computação Científica, Petrópolis 25651-075, Brazil

* Correspondence: earantes@lncc.br

check for updates

Citation: Paixão, E.A.; Barros, L.R.C.; Fassoni, A.C.; Almeida, R.C. Modeling Patient-Specific CAR-T Cell Dynamics: Multiphasic Kinetics via Phenotypic Differentiation. *Cancers* **2022**, *14*, 5576. https://doi.org/10.3390/cancers14225576

Academic Editor: Antonio Curti, Alessandro Isidori, Giuseppe Lo Russo and Marina Chiara Garassino

Received: 23 September 2022
Accepted: 7 November 2022
Published: 14 November 2022

Publisher's Note: MDPI stays neutral with regard to jurisdictional claims in published maps and institutional affiliations.

Copyright: © 2020 by the authors. Licensee MDPI, Basel, Switzerland. This article is an open access article distributed under the terms and conditions of the Creative Commons Attribution (CC BY) license (https://creativecommons.org/licenses/by/4.0/).

This Supplementary Material (SM) is organized as follows. Section 1 shows details about the experimental data obtained from [1–3] and unit transformations. Section 2 describes the method used for calibrating the model. Section 3 provides the fits obtained in a linear scale for all patients and the corresponding parameter values used in the final simulations. Finally, we present the values of the cellular kinetic parameters that were used to establish relationship with patient responses in Table S6.

1. Experimental Data

In vivo CAR-T cell data in the peripheral blood (PB) from different patients were obtained from [1–3]. Specifically, data from [2,3], measured in copies/μg DNA, were obtained directly from the corresponding supplementary materials while data from [1], presented in cells/μL, were extracted using the software WebPlotDigitizer [4] as shown in Table S1. Here, we make the units compatible by using CAR-T cell counts. Following [5], 1 cell/μL corresponds to 5×10^8 cells by assuming that each patient has 5L of blood, 1% of cells are in the PB, and converting μL to L. On the other hand, there is no standard transformation from copies/μg DNA to cell counts in the literature, although Mueller et al. [6] reported that 1 ng DNA is equivalent to 158.7 cells. Based on this information, we consider that 1 copy/μg DNA corresponds to approximately 10^5 cells.

2. Calibration Method

Our model was calibrated using a multi-step strategy based on the mechanisms underlying the multiphasic dynamics of the CAR-T cell therapy. Due to the relatively small quantity of data, the calibration process is not straightforward and requires a careful evaluation of the dynamics informed by the data. By performing an initial partition of the phases in the CAR-T kinetics, initial estimates of the parameters are obtained as described in the main text; these are sequentially updated until reaching reasonable fitting for the total CAR-T cells on a logarithmic scale. Afterward, the estimates are improved on a linear scale. The main steps of our calibration strategy encompass:

1. Analyze the dynamics informed by the data of the total CAR-T cells on a logarithmic scale and partition them in time as a function of the phases: distribution, expansion, contraction, and persistence.
2. Fit an exponential curve to each phase. For the expansion phase, we first try to fit the interior data points, excluding the endpoints, which mark the distribution and contraction phases. If there are not enough data points (at least two data points), we add the endpoint of the distribution phase. For patients with no data points in the distribution phase, we add the endpoint of the expansion phase.

3. Set the values of dominant parameters at each phase:
   - *Distribution phase:* $\beta = m_d$.
   - *Expansion phase:* $r_{min} + p_1 - \xi = m_e$. By additionally assuming a small initial value of $r_{min} = 1.0 \times 10^{-3}$ day^{-1} for the basal rate of clonal expansion and $\xi = \delta$, we get an initial estimate for p_1 .
   - *Contraction phase:* $\delta = m_c$.
   - *Persistence phase:* $\mu = m_p$.
4. Set $p_3 = 1.0$ and $p_2^{p_3} = 1/(\text{peak-day})^{p_3}$, in which the peak-day is the day with the highest recorded number of CAR-T cells. This assumption implies a progressive decrease in the CAR-T expansion rate, which becomes equal to $r_{min} + p_1/2$ at the peak-day.
5. Run the first model simulation after assigning initial values to the other parameters. Some of them were obtained from the literature and are given by: $\theta = 6.0 \times 10^{-6}$ (cell·day)$^{-1}$ [7]; $r = 0.176$ day^{-1}, $1/b = 2.0 \times 10^{12}$ cells, $\gamma = 2.25$ day^{-1}, $\vartheta = 0.305$ [8]. We additionally set $\eta = 1.0 \times 10^{-5}$ day^{-1}, $\epsilon = 1.0 \times 10^{-3}$ day^{-1}, $\lambda = 0.1$ day^{-1}, $A = 1.0$ cell, and $\alpha = 5.5 \times 10^{-7}$ (cell·day)$^{-1}$.
6. Improve the fitting by running a series of new simulations with new parameter values, starting by changing the values defined in the previous step. Other modifications should be done taking into account the following issues: (a) η should be changed so that the initial dynamics capture the first data point of the expansion phase, keeping the number of engrafted cells greater than or equal to one cell ($EC \geq 1$ cell); (b) the time of the CAR-T peak shifts to the left or right by increasing or decreasing, respectively, the value of A; (c) p_1, r_{min}, and ξ should be changed while keeping $m_e = p_1 + r_{min} - \xi$. If it does not work, they may be changed independently, noting that an increase in the values of p_1 and r_{min} leads to an increase in the CAR-T expansion; (d) an increase in the values of r_{min} can also make the contraction phase smoother and increase persistence; (e) λ and ϵ should be changed to adjust the peak values of memory and exhausted cells, which ultimately leads to a better fitting of the contraction and persistence phases.
7. If the fit is good enough, fine-tune the parameter values mainly to adjust the peak of total CAR-T cells in a linear scale. Otherwise, go back to step (i) and try a new data partition. As the distribution and expansion phases are, in general, well defined, the focus is to try a better separation between the contraction and persistence phases.

Our multi-step strategy led to patient-specific parameter values that fitted well to the various diseases and scenarios considered in this work. With the availability of more *in vivo* data encompassing the dynamics of both CAR-T cell phenotypes and tumor evolution, this process can be significantly improved. To further investigate if the dominant parameters at each phase of the CAR-T kinetics actually provide good approximations to the assigned model parameters, we compare the initial estimates (Y_{in}^i) with the final ones (Y_{out}^i), after calibration, through the root mean square error (RMSE):

$$RMSE = \sqrt{\frac{1}{n}\sum_{i=1}^{n}(Y_{out}^i - Y_{in}^i)^2},$$

in which n is the total number of patients considered in this work. The same error measure is also used in this work to compare observed (Y_{in}^i) and predicted (Y_{out}^i) CAR-T cells.

Figure S1 shows the RMSE values for each CAR-T phase for all patient data selected from 3 studies [1–3]. The persistence phase displays the smallest error due to the dominant presence of memory CAR-T cells in the later times of the dynamics. On the other hand, the highest error occurs in the expansion phase, as expected, due to the interplay among the effector, exhausted, and memory CAR-T cell populations.

3. Linear Scale Simulation Results

3.1. Patient Data from Liu et al.

The estimated parameter values and the corresponding model simulation (linear scale) for each patient are presented in Table S2 and Figure S2, respectively. Overall, model simulations show good agreement with the available data, independently of the disease, although smaller errors are observed in patients with DLBCL and pediatric ALL. RMSE between the predicted and observed data of total CAR-T cells for each disease is shown in Figure S6.

3.2. Patient Data from Brudno et al. and Porter et al.

The estimated parameter values and the corresponding model simulation (linear scale) for each patient are presented in Table S3 and Figure S3, respectively. Our model captured total CAR-T dynamics and patient outcomes. Figure S3 also displays details of the dynamics of cancer cells in later times, up to the time of the last follow-up. At this time, our model indicates that all CR patients have tumor burden at undetectable levels. Of note, patient B12 exhibits limit cycle oscillations and patient P1 has an extremely slow growth of cancer cells while the other patients have faster rates. All PR patients present similar behavior, although their tumor burdens reach measurable values at the last follow-up time. The model simulations also agreed with the disease relapse presented by the SD patients. A remarkable delay occurs in the early dynamics of cancer cells in SD patients, most evident in patient B2, who exhibits the later CAR-T cell peak and longest distribution phase (see Figure 4 - main text).

As mentioned before, the proposed model generated clinically undetectable cyclic behavior for some patients. We highlight the dynamics of patient B12 in Figure S4. Shortly after the infusion of CAR-T cells, the tumor cell population undergoes a strong decay. Around day 20, tumor cells resume growth until they start to behave cyclically from day 200. Total CAR-T cells keep decreasing after peaking, but effector cells grow back stimulated by the tumor until they reach the oscillatory behavior resulting from the interplay with the tumor cells. Among CAR-T cell phenotypes, memory cells exhibit cycles with the smallest amplitude.

Model simulations up to 10 years are shown in Figure S5 for the decade-long patients P1 and P2. Using data covering 4 years of measurements after therapy, Figures S5a and S5c show that model predictions agree with patient outcomes. The later dynamics of patient P2 indicate a limit cycle in which the populations of CAR-T and cancer cells alternate growth and decay at undetectable levels. We also re-calibrated the model for patient P2 including data extracted from [9] and shown in Table S4. The patient outcome remained tumor-free and is accompanied by greater persistence of CAR-T cells (Figure S5b).

By observing the cellular dynamics of the 24 patients considered in this study (see Figures 3 and 4 - main text), we distinguish two time points, t_1 and t_2, in the dynamics of CAR-T cells after the peak that mark transitions in the abundance of CAR-T cell phenotypes. When $t < t_1$, there is a predominance of effector cells, the exhausted cells become dominant after t_1, and from t_2 there is a dominance of memory cells. The time lag between t_1 and t_2 is patient-dependent and directly impacts the characterization of the dynamics. Patients 3, 27, and B5 present a greater region dominated by exhausted cells than patients 28, 30, and B20. Some patients display $t_1 \approx t_2$, such as B11, meaning that there is no dominance of exhausted cells during the CAR-T cell dynamics. Patient B2 does not exhibit a detectable t_2 point due to the formation of the memory pool at undetectable levels. Patient P2 also does not exhibit time t_2 due to a sharp decrease of the exhausted cells so that only memory cells are left after t_1. Overall, the characterization of t_1 and t_2 for the analyzed patient cohort did not show any correlation with long-term therapy responses.

In Table S6, the values of each evaluated kinetic parameter are presented to patients together with the corresponding recorded outcomes. The area under the concentration-time curve (AUC) was calculated using the trapezoidal rule.

Table S1. Patient data extracted from [1] whose the last follow-up responses (interval from infusion to the last follow-up in days) were either complete remission (CR), partial remission (PR), or stable disease (SD). The data were extracted using the software WebPlotDigitizer [4]. Zero counts in cells/μL were indicated as 2.5×10^6 cells (detection threshold), meaning that any value below the threshold is plausible. Doses were reported in cells/kg and were converted to cell counts considering that each patient weighs an average of 60 kg.

Days after infusion	CAR-T cells/μL	Total CAR-T cell counts
Patient 2 (DLBCL) — CAR-T dose = 4.2×10^7 cells — SD(30)		
3	0	2.5×10^6
12	9.1892	4.5946×10^9
14	9.1892	4.5946×10^9
20	0	2.5×10^6
Patient 5 (CLL) — CAR-T dose = 6.0×10^7 cells — CR(900+)		
5	0	2.5×10^6
9	27.0786	1.35393×10^{10}
12	30.4494	1.52247×10^{10}
14	14.7191	7.35955×10^9
Patient 10 (MCL) — CAR-T dose = 4.68×10^8 cells cells — SD(60)		
6	1.6216	8.1080×10^8
8	31.8919	1.594595×10^{10}
9	40.5405	2.027025×10^{10}
Patient 11 (CLL) — CAR-T dose = 1.86×10^8 cells cells — PR(540+)		
4	0	2.5×10^6
6	9.1011	4.55055×10^9
7	205.7303	1.0286515×10^{11}
8	77.6404	3.88202×10^{10}
11	3.4831	1.74155×10^9
13	0.1123	5.615×10^7
Patient 12 (ALL Ph+) — CAR-T dose = 3.12×10^8 cells — MRD$^-$ CR(480+)		
5	0.1123	5.615×10^7
7	33.8202	1.69101×10^{10}
9	14.7191	7.35955×10^9
12	0.1123	5.615×10^7
14	0.1123	5.615×10^7
16	0	2.5×10^6
Patient 15 (ALL Ph-neg) — CAR-T dose = 4.14×10^8 cells — MRD$^-$ CR(90)		
5	14.7191	7.35955×10^9
7	63.0337	3.151685×10^{10}
8	28.2022	1.41011×10^{10}
Patient 18 (DLBCL) — CAR-T dose = 1.86×10^8 cells — SD(60)		
5	5.4054	2.7027×10^9
7	40.5405	2.027025×10^{10}
9	13.5135	6.75675×10^9
12	11.3513	5.67565×10^9
Patient 20 (ALL Ph-neg) — CAR-T dose = 2.52×10^8 cells — MRD$^-$ CR(90+)		
4	9.1011	4.55055×10^9
6	260.7865	1.3039325×10^{11}
8	29.3258	1.46629×10^{10}
11	1.2359	6.1795×10^8

DLBCL — diffuse large–B-cell lymphoma. CLL — chronic lymphocytic leukemia. MCL — mantle cell lymphoma. ALL Ph+ —Philadelphia chromosome-positive acute lymphoblastic leukemia. ALL Ph-neg – Philadelphia chromosome-negative acute lymphoblastic leukemia.

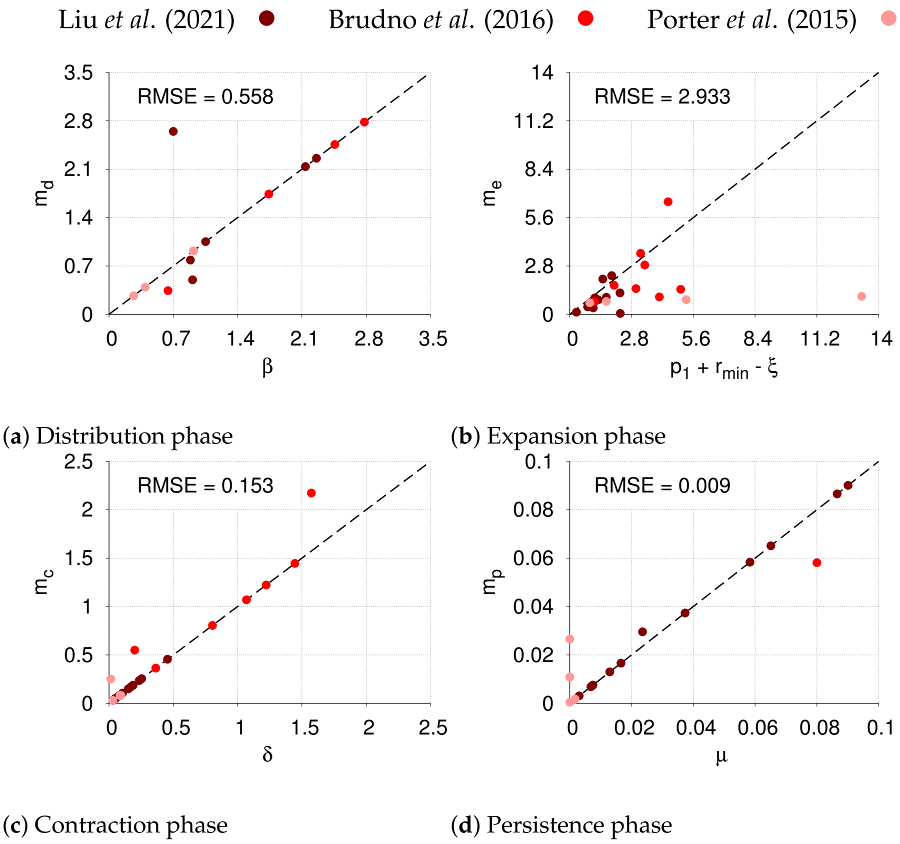

Figure S1. Root mean square error (RMSE) between the approximations of dominant parameters at each phase of the CAR-T dynamics and the assigned model parameters. It includes data from different references [1–3] as indicated by the color of the dots.

Table S2. Calibrated parameter values, in appropriate units (a.u.), used in the simulations for patient data obtained from [3]. Patients were divided into four groups according to the reported disease: diffuse large–B-cell lymphoma (DLBCL), pediatric and adult acute lymphoblastic leukemia (ALL), and chronic lymphocytic leukemia (CLL). In all simulations, we set the initial tumor burden equal to 1.0×10^7 cells and a single CAR-T dose. As the doses for patients with ALL were not informed, we used for them a dose of 1.0×10^8 CAR-T cells corresponding to the median total dose for patients weighing > 50 Kg reported in [10]. The peak-day observed in the experimental data is also informed, which was used to determine the parameter p_2 ($p_2^{p_3} = 1/(\text{peak-day})^{p_3}$). The approximated total number of engrafted CAR-T cells (EC) is evaluated for each patient using equation (9). Parameters whose values were the same for all patients are: $\theta = 6.0 \times 10^{-6}$, $\alpha = 5.5 \times 10^{-7}$, $r = 1.76 \times 10^{-1}$, $1/b = 2.0 \times 10^{12}$, $\vartheta = 3.05 \times 10^{-1}$, and $a = 1.0 \times 10^3$.

Parameter	DLBCL			ALL Pediatric			ALL Adult			CLL		
	**Patient 27**	**Patient 28**	**Patient 30**	**Patient A**	**Patient J**	**Patient L**	**Patient 1**	**Patient 3**	**Patient 7**	**Patient UPN09**	**Patient UPN10**	**Patient UPN17**
β	2.1384	1.0512	2.2586	8.0×10^{-1}	1.0×10^{-1}	1.0×10^{-3}	1.0×10^{-1}	2.1	1.0×10^{-1}	9.0938×10^{-1}	7.0×10^{-1}	8.8583×10^{-1}
η	3.5×10^{-1}	5.4×10^{-2}	1.0×10^{-1}	2.0	1.0×10^{-1}	6.0	3.0×10^{-3}	1.0×10^{-1}	7.0×10^{-3}	8.0×10^{-6}	4.0×10^{-5}	5.0×10^{-3}
r_{min}	1.0×10^{-3}	1.0×10^{-3}	1.0×10^{-3}	1.0×10^{-1}	6.0×10^{-1}	4.5×10^{-2}	3.8×10^{-1}	5.0×10^{-2}	1.5×10^{-1}	5.0×10^{-1}	5.0×10^{-1}	1.0×10^{-4}
p_1	2.409202	1.750021	2.625285	9.021974×10^{-1}	5.0600965×10^{-1}	5.489287	1.511182	2.925191	1.200377	1.9007101	9.9×10^{-3}	1.342692
p_2	1.5849×10^{-5}	7.5389×10^{-25}	5.5944×10^{-8}	3.8787×10^{-2}	4.4194×10^{-2}	7.3597×10^{-2}	6.7268×10^{-3}	1.1220×10^{-2}	4.9385×10^{-58}	6.25×10^{-2}	1.8518×10^{-2}	1.0×10^{-16}
p_3	4.8	3.1×10	7.6	1.2	1.5	1.05	1.95	1.95	5.0×10	1.0	1.0	1.6×10
A	1.0×10^{-1}	5.0×10	1.0×10^{-4}	1.0×10^{-6}	1.0×10^{-1}	2.0×10^{-5}	1.0×10^{-3}	3.0×10^{-3}	2.0×10^{-7}	5.0×10^{-3}	5.5×10^{-13}	3.0×10^{-2}
ξ	1.27078	2.5476×10^{-1}	1.33758	1.9204×10^{-1}	4.2255×10^{-2}	3.24424	2.3446×10^{-1}	1.077018	3.7564×10^{-1}	1.23133×10^{-1}	2.0464×10^{-1}	2.3576×10^{-1}
ϵ	2.0×10^{-2}	6.0×10^{-2}	2.0×10^{-2}	3.0×10^{-3}	8.0×10^{-3}	1.6×10^{-3}	4.5×10^{-2}	2.0×10^{-2}	5.0×10^{-2}	1.3×10^{-3}	3.0×10^{-3}	5.0×10^{-3}
λ	1.0×10^{-1}	1.0×10^{-1}	1.0×10^{-1}	1.0×10^{-1}	1.0×10^{-1}	1.0×10^{-1}	1.0×10^{-1}	1.0×10^{-1}	1.0×10^{-1}	1.0×10^{-2}	2.0×10^{-2}	3.0×10^{-2}
μ	5.8352×10^{-2}	9.0101×10^{-2}	6.5105×10^{-2}	6.8474×10^{-3}	7.4965×10^{-3}	1.6579×10^{-2}	1.2962×10^{-2}	3.7325×10^{-2}	8.6577×10^{-2}	3.0541×10^{-3}	7.386×10^{-3}	2.3562×10^{-2}
δ	1.7078×10^{-1}	1.4976×10^{-1}	4.5473×10^{-1}	1.0504×10^{-1}	4.2255×10^{-2}	2.5424×10^{-1}	2.3446×10^{-1}	7.7018×10^{-2}	1.8664×10^{-1}	5.3133×10^{-2}	2.9642×10^{-2}	1.8576×10^{-1}
γ	2.25	2.25	2.25	2.25	2.25	2.25	2.25	2.25	2.25	2.25	1.0	2.25
**Total dose**	5.9895×10^7	9.2303×10^7	1.2767×10^8	1.0×10^8	1.0×10^8	1.0×10^8	1.0×10^8	1.0×10^8	1.0×10^8	1.7×10^8	5.61×10^8	1.03×10^8
**Peak-day**	10	6	9	15	8	12	13	10	14	16	54	10
EC	8.4244×10^6	4.5099×10^6	5.4129×10^6	7.1428×10^7	5.0×10^7	9.9983×10^7	2.9126×10^6	4.5454×10^6	6.5420×10^6	1.4955×10^3	3.2055×10^4	5.7811×10^5

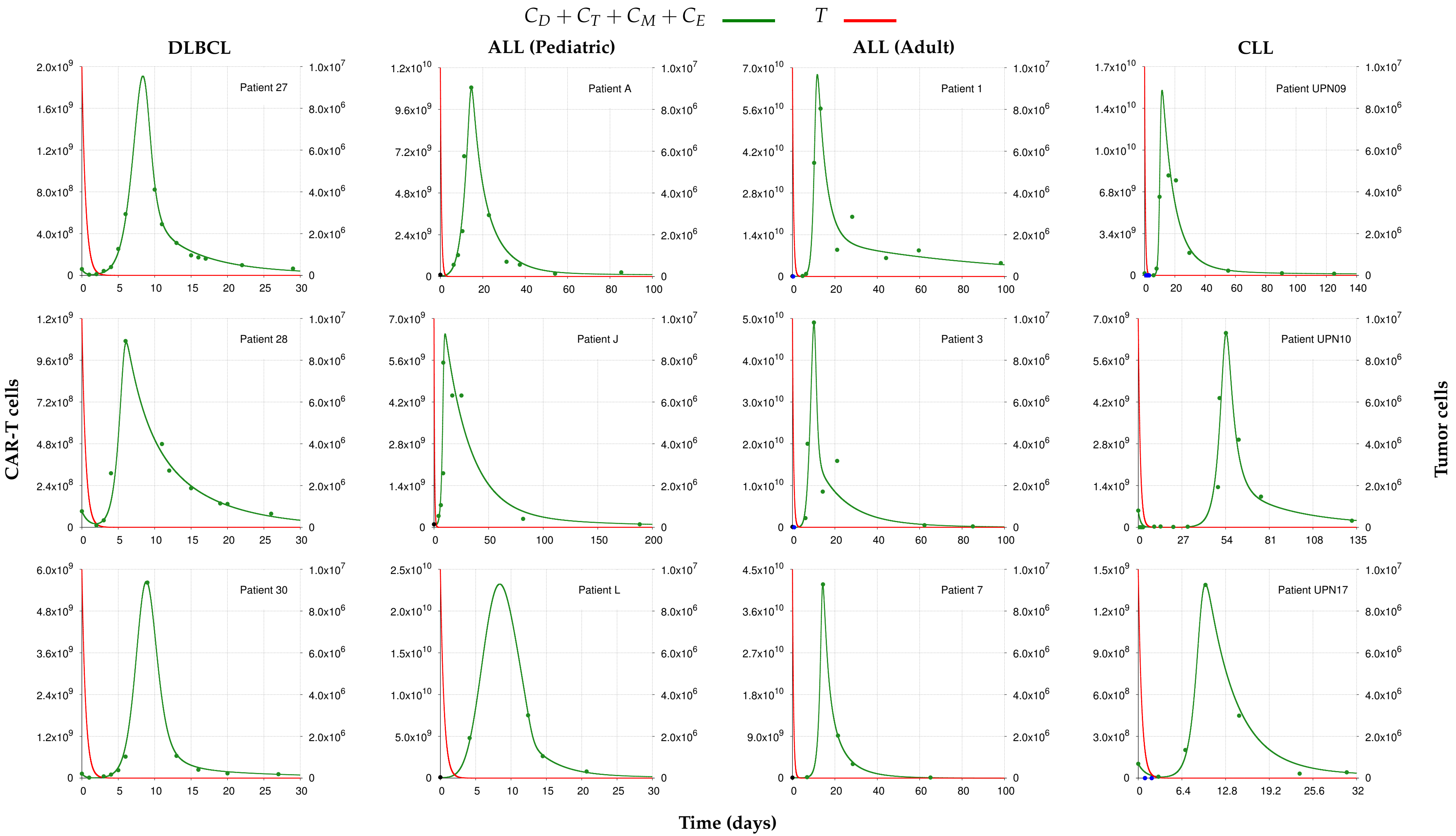

Figure S2. Model simulations fitted to the experimental data (●) from [3] on a linear scale. Each column corresponds to the dynamics of the total CAR-T cell population (▬) for different diseases (DLBCL, pediatric and adult ALL, and CLL) and different patients. Tumor cells (▬) decay exponentially due to the cytotoxic effect of CAR-T cells. The mean dose value of 1.0×10^8 cells (●) presented in [10] is used as a surrogate for the actual doses when not reported for patients with ALL. Data points in the clinically undetectable region (●) were not used for calibration and error calculation due to their high uncertainties. The quantification thresholds are summarized in Table S1 of [3].

Table S3. Calibrated parameter values, in appropriate units (a.u.), used in the simulations for patient data obtained from [1,2]. Patients were divided into three groups according to the reported response: CR — complete response, PR — partial response, and SD — stable disease. In all simulations, we set the initial tumor burden equal to 1.0×10^7 cells. A single dose was applied to patients from [1] and fractionated dose (10% on day 0, 30% on day 1, and 60% on day 2) to those from [2]. The peak-day observed in the experimental data is also informed, which was used to determine the parameter p_2 ($p_2^{p_3} = 1/(\text{peak-day})^{p_3}$). The approximated total number of engrafted CAR-T cells (EC) is evaluated for each patient using equation (9). Parameters whose values were the same for all patients are: $\alpha = 5.5 \times 10^{-7}$, $r = 1.76 \times 10^{-1}$, $1/b = 2.0 \times 10^{12}$, and $\vartheta = 3.05 \times 10^{-1}$.

Parameter	CR						PR			SD		
	**B15[1] - ALL MDR$^-$ CR(90)**	**B20[1] - ALL MDR$^-$ CR(90+)**	**B12[1] - ALL MDR$^-$ CR(480+)**	**B5[1] - CLL CR(900+)**	**P2[2] - CLL MDR$^-$ CR(1560+)**	**P1[2] - CLL MDR$^-$ CR(1590+)**	**P12[2] - CLL PR(180)**	**P22[2] - CLL PR(300)**	**B11[1] - CLL PR(540+)**	**B2[1] - DLBCL SD(30)**	**B10[1] - MCL SD(60)**	**B18[1] - DLBCL SD(60)**
β	2.0×10^{-1}	3.8	6.43×10^{-1}	1.7399	2.675×10^{-1}	9.1921×10^{-1}	3.9363×10^{-1}	1.0×10^{-3}	2.4577	2.7809	3.5	3.5
η	4.0×10^{-1}	4.0×10^{-1}	1.6×10^{-7}	5.0×10^{-8}	2.0×10^{-3}	1.0×10^{-8}	3.0×10^{-2}	5.0×10^{-1}	2.0×10^{-8}	1.0	2.0×10^{-2}	1.0×10^{-2}
r_{min}	1.0×10^{-3}	4.25×10^{-1}	2.3	1.0×10^{-3}	4.5×10^{-1}	3.03	6.0×10^{-1}	1.0×10^{-1}	1.0	1.631	1.0×10^{-2}	1.0×10^{-3}
p_1	2.50812	3.0351	2.7864	3.6	5.32453×10^{-1}	1.050917958×10	1.57	5.625	4.7146	6.5555	5.14	6.7495
p_2	3.2097×10^{-18}	5.8171×10^{-28}	7.6962×10^{-33}	1.3459×10^{-12}	3.2258×10^{-2}	2.7841×10^{-41}	7.7452×10^{-67}	4.0505×10^{-2}	1.4678×10^{-72}	4.3941×10^{-10}	1.8648×10^{-8}	1.2958×10^{-4}
p_3	2.07×10	3.5×10	3.8×10	1.1×10	1.0	8.5×10	5.0×10	1.184	8.5×10	8.4	8.1	4.6
A	1.0×10^{-6}	1.0×10	6.0×10^{-1}	6.0×10^{-2}	1.0×10^{-7}	8.1×10^{4}	1.0×10^{2}	1.0×10^{6}	9.5	1.0×10^{-1}	6.9×10^{3}	1.0×10^{4}
a	1.0×10^{3}	1.0×10^{3}	1.0×10^{3}	1.0×10^{3}	1.0×10^{4}	1.0×10^{3}	2.0×10^{6}	1.8×10^{6}	1.0×10^{3}	1.0×10^{4}	5.0×10^{5}	5.0×10^{6}
ξ	1.39427	1.4492	1.6858	3.8946×10^{-1}	7.298×10^{-2}	3.09366×10^{-1}	5.09828×10^{-1}	4.5151×10^{-1}	1.2584	3.1598	2.14908	2.68908
ϵ	8.0×10^{-4}	1.5×10^{-3}	1.0×10^{-4}	9.0×10^{-3}	4.1×10^{-3}	1.7×10^{-4}	5.5×10^{-4}	1.225×10^{-3}	4.1×10^{-4}	1.0×10^{-4}	1.0×10^{-5}	1.0×10^{-5}
λ	1.0×10^{-1}	1.0×10^{-1}	1.0×10^{-1}	3.0×10^{-1}	1.0×10^{-3}	3.0×10^{-2}	6.0×10^{-1}	2.5×10^{-3}	1.0×10^{-1}	8.0×10^{-1}	5.0×10^{-1}	4.0×10^{-1}
θ	6.0×10^{-6}	6.0×10^{-6}	6.0×10^{-6}	6.0×10^{-6}	6.0×10^{-6}	6.0×10^{-6}	5.0×10^{-7}	2.0×10^{-7}	6.0×10^{-6}	6.0×10^{-6}	6.0×10^{-6}	6.0×10^{-6}
μ	2.0×10^{-2}	2.0×10^{-2}	1.0×10^{-1}	1.0×10^{-2}	1.727×10^{-3}	4.758×10^{-5}	1.0×10^{-6}	1.0×10^{-5}	2.0×10^{-2}	4.0×10^{-2}	8.0×10^{-2}	8.0×10^{-2}
δ	8.0427×10^{-1}	1.0692	1.4458	3.6346×10^{-1}	2.9748×10^{-2}	8.5366×10^{-2}	9.252×10^{-2}	1.351×10^{-2}	1.2224	1.573	1.5	2.0×10^{-1}
γ	2.25	2.25	2.25	2.25	1.8	2.25	1.19	4.8×10^{-1}	2.25	3.69×10^{-1}	8.5×10^{-1}	1.2
**Total dose**	4.14×10^{8}	2.52×10^{8}	3.12×10^{8}	6.0×10^{7}	1.42×10^{7}	1.13×10^{9}	1.18×10^{8}	8.64×10^{7}	1.86×10^{8}	4.2×10^{7}	4.68×10^{8}	1.86×10^{8}
**Peak-day**	7	6	7	12	31	3	21	15	7	13	9	7
EC	2.76×10^{8}	2.4×10^{7}	7.7636×10	1.7242	1.0538×10^{5}	1.2293×10	8.3563×10^{6}	8.6227×10^{7}	1.5136	1.1108×10^{7}	2.6591×10^{6}	5.2991×10^{5}

Experimental data: [1] [1]; [2] [2].

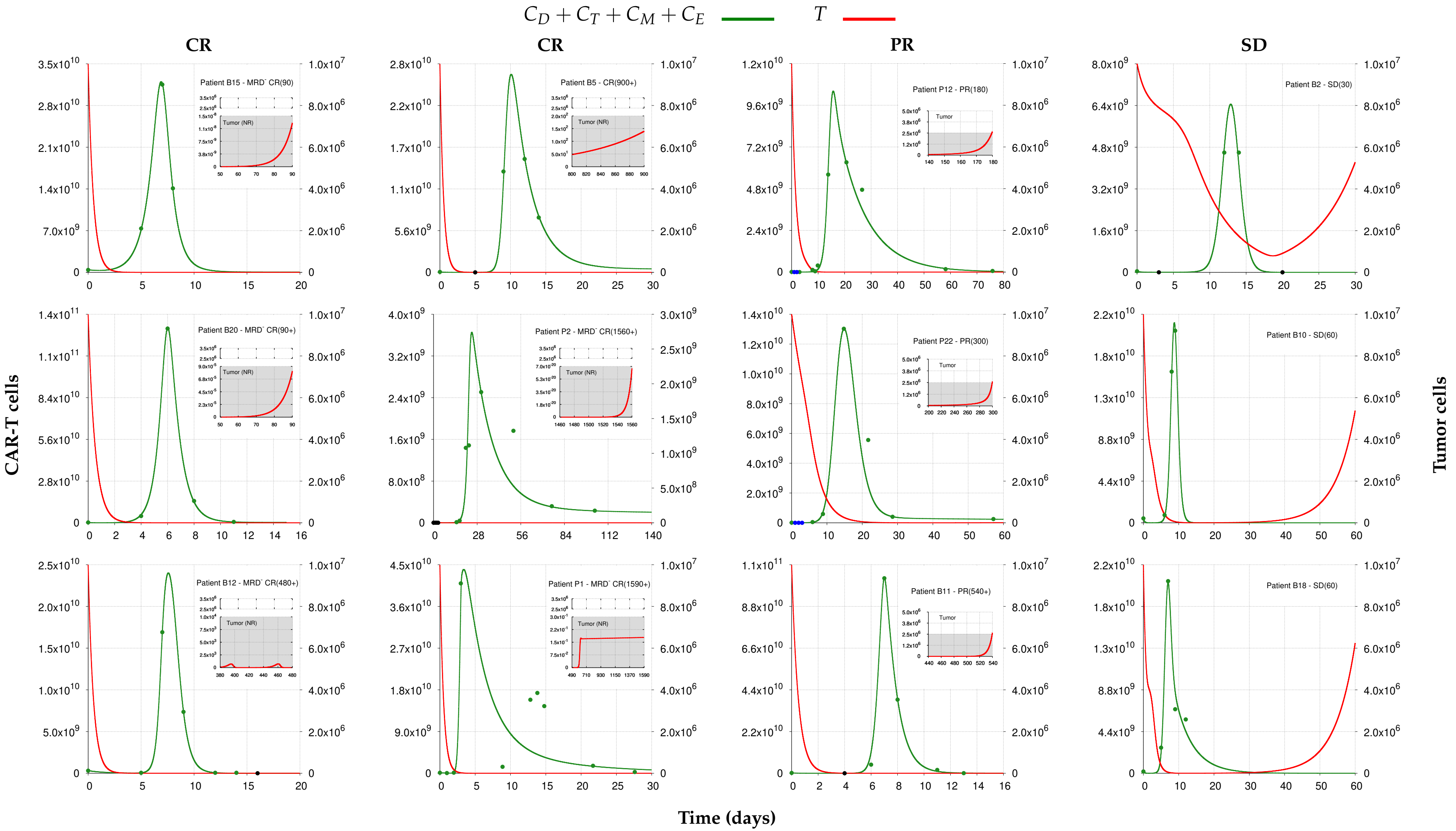

Figure S3. Model simulations fitted to the experimental data (●) from [1,2] on a linear scale. Each column corresponds to the dynamics of the total CAR-T cell population (▬) for different therapy responses at the last follow-up (interval from infusion to the last follow-up in days) (CR – complete response; PR – partial response; SD – stable disease) and different patients. Tumor cell populations (▬) presented several behaviors. Data points (●) may assume any value under the detection threshold ($\leq 2.5 \times 10^6$ cells), but some (●) were not used for calibration and error calculation of the model due to their greater uncertainty.

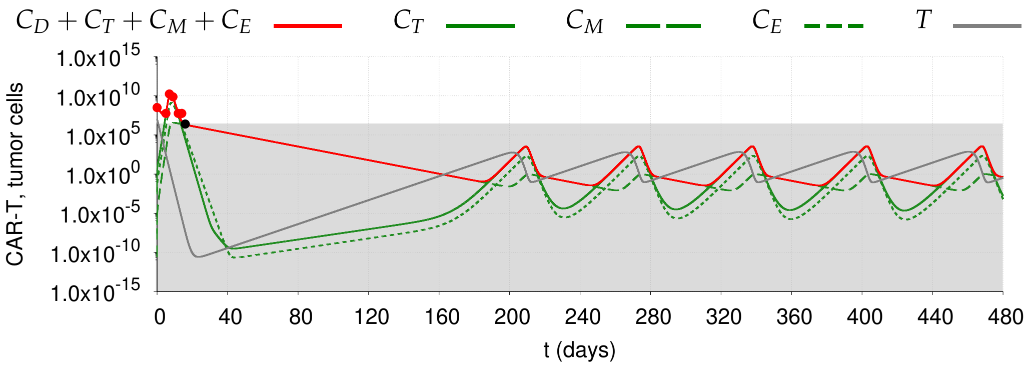

Figure S4. Simulated dynamics for patient B12 - MRD$^-$ CR(480+). Cyclic behavior of CAR-T and tumor cells is observed from day 200, with cell populations remaining below the detection threshold. Gray shadow represents the undetectable level ($\leq 2.5 \times 10^6$ cells). Experimental data (●) from [1] with points (●) indicating CAR-T cell population under the detection threshold ($\leq 2.5 \times 10^6$ cells).

Table S4. Data of the decade-long patients P1 and P2 were extracted from [9]. Data are reported in both copies/μg DNA and cell counts.

Days after infusion	CAR-T copies/μg DNA	Total CAR-T cell counts
Patient P1 (CLL) — CAR-T dose = 1.13×10^9 cells		
1683.9867	738.9467	7.3895×10^7
1780.8638	1265.2891	1.2653×10^8
1974.6179	1034.1857	1.0342×10^8
2954.1528	3709.7365	3.7097×10^8
3007.9734	8596.2011	8.5962×10^8
3255.5482	11633.0456	1.1633×10^9
3600.0000	9833.3326	9.8333×10^8
Patient 2 (CLL) — CAR-T dose = 1.42×10^7 cells		
1670.1992	290.9163	2.9092×10^7
1696.0159	254.5641	2.5456×10^7
1799.2829	332.4598	3.3246×10^7
1928.3665	464.1589	4.6416×10^7
2173.6255	332.4598	3.3246×10^7
2341.4343	118.1582	1.1816×10^7
2593.1474	464.1589	4.6416×10^7
3277.2908	379.9357	3.7993×10^7

Table S5. Calibrated parameter values, in appropriate units (a.u.), used in the new simulations for the decade-long patient P2, whose data were obtained from [2,9]. We set the initial tumor burden equal to 1.0×10^7 cells and the dose of 1.42×10^7 CAR-T cells given in the full 3-day split-dose regimen (10% on day 0, 30% on day 1, and 60% on day 2).

**Parameter**	β	η	r_{min}	p_1	p_2	p_3	A
**Estimated value**	2.675×10^{-1}	2.0×10^{-3}	4.0×10^{-1}	5.62453×10^{-1}	3.2258×10^{-2}	1.0	2.0×10^{-7}
**Parameter**	a	ξ	ϵ	λ	θ	α	
**Estimated value**	1.0×10^{4}	3.8748×10^{-2}	1.0×10^{-3}	8.0×10^{-3}	6.0×10^{-6}	5.5×10^{-7}	
**Parameter**	μ	δ	r	b	γ	ϑ	
**Estimated value**	1.827×10^{-4}	2.9748×10^{-2}	1.76×10^{-1}	5.0×10^{-13}	1.8	3.05×10^{-1}	

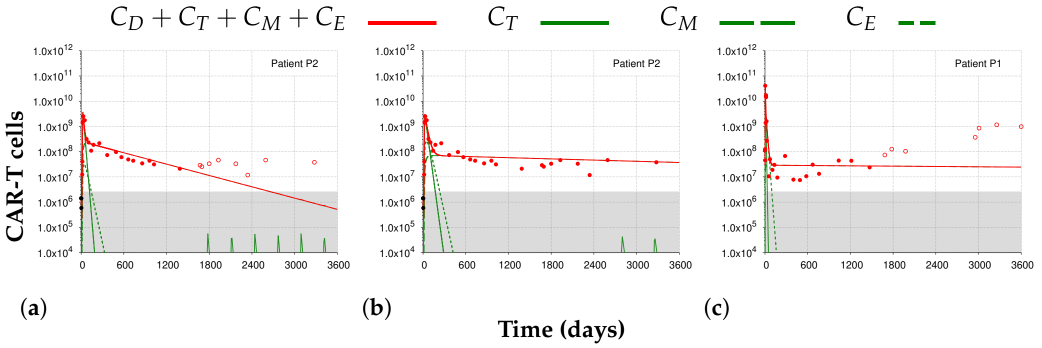

Figure S5. Model simulations for the decade-long patients P1 and P2, whose data are shown by red dots. Filled red dots indicate the experimental data used for model calibration in panels (a) and (c), covering 4 years of measurements after therapy, while the empty red dots were disregarded. In panel (b), simulations were performed by using all available data. Panels (a) and (b) show remarkable differences in the persistence of the total CAR-T cells.

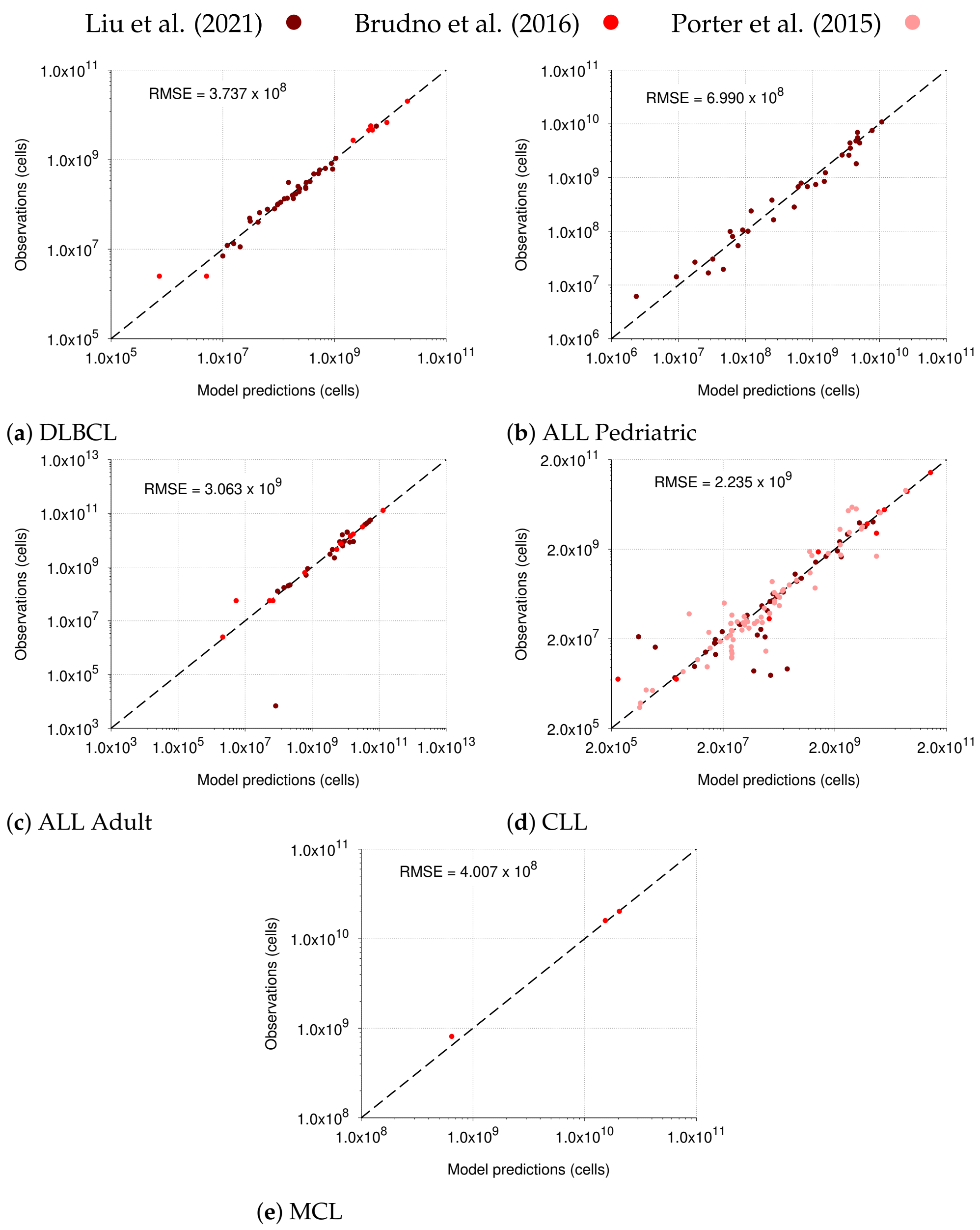

Figure S6. Root mean square error (RMSE) between the predicted and observed data of total CAR-T cells. Each panel corresponds to a different disease (diffuse large-B-cell lymphoma – DLBCL; pediatric and adult lymphoblastic leukemia – ALL; chronic lymphocytic leukemia – CLL; mantle cell lymphoma –MCL) and includes data from different references [1–3] as indicated by the color of the dots.

Table S6. Cellular kinetic parameters assessed for each patient with complete response (CR), partial response (PR), or stable disease (SD).

Kinetic Parameter	CR						PR			SD		
	**B15[1] - ALL MDR^- CR(90)**	**B20[1] - ALL MDR^- CR(90+)**	**B12[1] - ALL MDR^- CR(480+)**	**B5[1] - CLL CR(900+)**	**P2[2] - CLL MDR^- CR(1560+)**	**P1[2] - CLL MDR^- CR(1590+)**	**P12[2] - CLL PR(180)**	**P22[2] - CLL PR(300)**	**B11[1] - CLL PR(540+)**	**B2[1] - DLBCL SD(30)**	**B10[1] - MCL SD(60)**	**B18[1] - DLBCL SD(60)**
AUC0-28, cell × day	4.1750×10^{10}	1.2064×10^{11}	2.4296×10^{10}	1.1812×10^{11}	2.1965×10^{10}	2.2540×10^{11}	9.8309×10^{10}	1.0152×10^{11}	8.0239×10^{10}	9.8328×10^{9}	2.6858×10^{10}	4.6083×10^{10}
% AUC0-28$_{C_D}$	0.8263	0.0249	0.9985	0.0292	0.0240	0.0546	0.0283	0.0170	0.0471	0.0565	0.2475	0.0575
% AUC0-28$_{C_T}$	87.1350	89.2577	92.5219	50.8368	98.2621	76.8757	18.0077	96.0636	91.8060	66.2497	74.8114	33.6402
% AUC0-28$_{C_M}$	1.2047	2.3693	0.0802	7.2719	1.3955	0.2852	0.1304	1.2187	0.6366	0.0005	0.0040	0.0021
% AUC0-28$_{C_E}$	10.8340	8.3481	6.3993	41.8621	0.3184	22.7845	81.8335	2.7006	7.5103	33.6934	24.9371	66.3002
AUC0-60, cell × day	4.2197×10^{10}	1.2310×10^{11}	2.4298×10^{10}	1.3068×10^{11}	6.2526×10^{10}	2.3529×10^{11}	1.3120×10^{11}	1.1043×10^{11}	8.0711×10^{10}	9.8328×10^{9}	2.6858×10^{10}	4.6534×10^{10}
% AUC0-60$_{C_D}$	0.8176	0.0244	0.9984	0.0264	0.0084	0.0523	0.0212	0.0156	0.0469	0.0565	0.2475	0.0569
% AUC0-60$_{C_T}$	86.2111	87.4721	92.5110	45.9493	88.7204	73.6586	13.4935	88.6030	91.2687	66.2496	74.8113	33.3142
% AUC0-60$_{C_M}$	2.2521	4.3225	0.0920	16.0977	9.6678	0.6679	0.3351	4.0162	1.2181	0.0005	0.0041	0.0022
% AUC0-60$_{C_E}$	10.7192	8.1811	6.3986	37.9266	1.6034	25.6213	86.1502	7.3651	7.4663	33.6934	24.9371	66.6267
AUC0-90, cell × day	4.2423×10^{10}	1.2434×10^{11}	2.4299×10^{10}	1.3924×10^{11}	7.2389×10^{10}	2.3673×10^{11}	1.3316×10^{11}	1.1660×10^{11}	8.0949×10^{10}	9.8328×10^{9}	2.6858×10^{10}	4.6534×10^{10}
% AUC0-90$_{C_D}$	0.8132	0.0241	0.9984	0.0248	0.0073	0.0519	0.0209	0.0148	0.0467	0.0565	0.2475	0.0569
% AUC0-90$_{C_T}$	85.7536	86.6001	92.5105	43.1262	80.4233	73.2095	13.2949	83.9302	91.0006	66.2497	74.8113	33.3142
% AUC0-90$_{C_M}$	2.7709	5.2762	0.0925	21.2526	17.4253	1.0310	0.5491	6.2295	1.5082	0.0005	0.0041	0.0022
% AUC0-90$_{C_E}$	10.6623	8.0995	6.3986	35.5964	2.1441	25.7076	86.1351	9.8255	7.4444	33.6934	24.9371	66.6267
t_{peak}, day	6.8936	6.0157	7.5721	10.1003	24.5411	3.3808	15.7029	14.8725	7.0074	12.8881	8.7598	6.9961
$C(t_{peak})$, cell	3.1996×10^{10}	1.3090×10^{11}	2.4008×10^{10}	2.6580×10^{10}	3.6547×10^{9}	4.3975×10^{10}	1.0408×10^{10}	1.3018×10^{10}	1.0308×10^{11}	6.4479×10^{9}	2.1105×10^{10}	2.0105×10^{10}
$C_D(t_{peak})$, cell	6.6174×10^{6}	2.6825×10^{-3}	2.3968×10^{6}	1.3998	1.9052×10^{3}	5.0517×10^{6}	1.5236×10^{4}	5.0180×10^{3}	6.1667	2.8885×10^{-14}	1.9010×10^{-5}	4.0257×10^{-3}
$C_T(t_{peak})$, cell	2.9811×10^{10}	1.2476×10^{11}	2.2979×10^{10}	2.0493×10^{10}	3.6032×10^{9}	4.3162×10^{10}	3.8528×10^{9}	1.2873×10^{10}	9.9358×10^{10}	4.4919×10^{9}	1.6713×10^{10}	1.3716×10^{10}
$C_M(t_{peak})$, cell	3.1600×10^{7}	1.4905×10^{8}	1.7777×10^{6}	2.2249×10^{8}	4.1845×10^{7}	4.5165×10^{6}	6.9536×10^{6}	4.3429×10^{7}	2.2696×10^{7}	3.4334×10^{4}	1.3808×10^{5}	1.2167×10^{5}
$C_E(t_{peak})$, cell	2.1468×10^{9}	5.9944×10^{9}	1.0250×10^{9}	5.8655×10^{9}	9.6922×10^{6}	8.0378×10^{8}	6.5484×10^{9}	1.0149×10^{8}	3.7016×10^{9}	1.9559×10^{9}	4.3919×10^{9}	6.3892×10^{9}
$T(t_{peak})$, cell	6.2321	4.0265×10	1.5360	1.1230×10^{-2}	1.0841×10^{-8}	9.4375×10^{3}	4.4388×10	2.5286×10^{5}	6.3599	2.1509×10^{6}	6.2955×10^{4}	4.9228×10^{4}
t_1, day	9.7778	9.5984	12.3922	11.7264	102.8099	11.7893	14.8152	27.1194	13.1751	14.5770	10.6339	7.6529
t_2, day	13.4598	10.6782	13.8163	21.2374	NA	67.0912	90.0001	92.2745	12.7001	c	17.5925	c
t_{TR}, day	468.6538	567.4207	a	1143.9747	a	b	179.6661	299.7583	539.6457	25.7534	55.6440	54.7998
$C(t_{TR})$, cell	2.5073×10^{-5}	1.0332×10^{-6}	NA	2.4286×10^{-1}	NA	NA	5.8402×10^{5}	5.6166×10^{6}	1.6723×10^{-4}	8.5618×10	2.5442×10^{-8}	8.4935×10^{5}
$C_D(t_{TR})$, cell	3.1387×10^{-114}	0.0	NA	0.0	NA	NA	1.0396×10^{-26}	5.1837×10^{-59}	0.0	2.1654×10^{-35}	4.0396×10^{-77}	5.4218×10^{-76}
$C_T(t_{TR})$, cell	1.0206×10^{-11}	9.5277×10^{-10}	NA	5.5780×10^{-7}	NA	NA	9.0543×10^{2}	5.9082×10^{4}	7.9949×10^{-5}	1.2407×10^{-4}	3.0638×10^{-16}	4.9567×10^{-18}
$C_M(t_{TR})$, cell	6.8317×10^{-16}	1.1594×10^{-13}	NA	3.9343×10^{-10}	NA	NA	5.6978×10^{2}	1.4223×10^{5}	2.5015×10^{-9}	1.1135×10^{-9}	2.8253×10^{-22}	4.7728×10^{-24}
$C_E(t_{TR})$, cell	2.5073×10^{-5}	1.0322×10^{-6}	NA	2.4286×10^{-1}	NA	NA	5.8255×10^{5}	5.4153×10^{6}	8.7275×10^{-5}	8.5618×10	2.5442×10^{-8}	8.4935×10^{5}

Experimental data: [1] [1]; [2] [2]. a – Limit cycle at undetectable levels; b – No tumor recurrence in 20,000 days; c – Memory cell counts lower than the exhausted cell counts; NA – Not applicable.

References

1. Brudno, J.N.; Somerville, R.P.; Shi, V.; Rose, J.J.; Halverson, D.C.; Fowler, D.H.; Gea-Banacloche, J.C.; Pavletic, S.Z.; Hickstein, D.D.; Lu, T.L.; et al. Allogeneic T Cells That Express an Anti-CD19 Chimeric Antigen Receptor Induce Remissions of B-Cell Malignancies That Progress After Allogeneic Hematopoietic Stem-Cell Transplantation Without Causing Graft-Versus-Host Disease. *J. Clin. Oncol.* **2016**, *34*, 1112–1121. https://doi.org/10.1200/JCO.2015.64.5929.
2. Porter, D.L.; Hwang, W.T.; Frey, N.V.; Lacey, S.F.; Shaw, P.A.; Loren, A.W.; Bagg, A.; Marcucci, K.T.; Shen, A.; Gonzalez, V.; et al. Chimeric antigen receptor T cells persist and induce sustained remissions in relapsed refractory chronic lymphocytic leukemia. *Sci. Transl. Med.* **2015**, *7*, 303ra139. https://doi.org/10.1126/scitranslmed.aac5415.
3. Liu, C.; Ayyar, V.S.; Zheng, X.; Chen, W.; Zheng, S.; Mody, H.; Wang, W.; Heald, D.; Singh, A.P.; Cao, Y. Model-Based Cellular Kinetic Analysis of Chimeric Antigen Receptor-T Cells in Humans. *Clin. Pharmacol. Ther.* **2021**, *109*, 716–727. https://doi.org/10.1002/cpt.2040.
4. Rohatgi, A. WebPlotDigitizer, 2020. Version 4.5. Available online: https://apps.automeris.io/wpd/ (accessed on 13 December 2021).
5. Kimmel, G.J.; Locke, F.L.; Altrock, P.M. Response to CAR T cell therapy can be explained by ecological cell dynamics and stochastic extinction events. *bioRxiv* **2020**. https://doi.org/10.1101/717074.
6. Mueller, K.; Maude, S.; Porter, D.; Frey, N.; Wood, P.; Han, X.; Waldron, E.; Chakraborty, A.; Awasthi, R.; Levine, B.; Melenhorst, J.; Grupp, S.; June, C.; Lacey, S. Cellular kinetics of CTL019 in relapsed/refractory B-cell acute lymphoblastic leukemia and chronic lymphocytic leukemia. *Blood* **2017**, *130*, 2317–2325. https://doi.org/10.1182/blood-2017-06-786129.
7. Barros, L.R.C.; Paixão, E.A.; Valli, A.M.P.; Naozuka, G.T.; Fassoni, A.C.; Almeida, R.C. CARTmath—A Mathematical Model of CAR-T Immunotherapy in Preclinical Studies of Hematological Cancers. *Cancers* **2021**, *13*, 2941. https://doi.org/10.3390/cancers13122941.
8. Owens, K.; Bozic, I. Modeling CAR T-Cell Therapy with Patient Preconditioning. *Bull. Math. Biol.* **2021**, *83*, 42–36. https://doi.org/10.1007/s11538-021-00869-5.
9. Melenhorst, J.J.; Chen, G.M.; Wang, M.; Porter, D.L.; Chen, C.; Collins, M.A.; Gao, P.; Bandyopadhyay, S.; Sun, H.; Zhao, Z.; et al. Decade-long leukaemia remissions with persistence of CD4+ CAR T cells. *Nature* **2022**, *602*, 1476–4687. https://doi.org/10.1038/s41586-021-04390-6.
10. Stein, A.M.; Grupp, S.A.; Levine, J.E.; Laetsch, T.W.; Pulsipher, M.A.; Boyer, M.W.; August, K.J.; Levine, B.L.; Tomassian, L.; Shah, S.; et al. Tisagenlecleucel Model-Based Cellular Kinetic Analysis of Chimeric Antigen Receptor–T Cells. *CPT Pharmacometrics Syst. Pharmacol.* **2019**, *8*, 285–295. https://doi.org/10.1002/psp4.12388.
